# Immunohistochemical Monitoring of Wound Healing in Antibiotic Treated Buruli Ulcer Patients

**DOI:** 10.1371/journal.pntd.0002809

**Published:** 2014-04-24

**Authors:** Arianna Andreoli, Marie-Thérèse Ruf, Ghislain Emmanuel Sopoh, Peter Schmid, Gerd Pluschke

**Affiliations:** 1 Swiss Tropical and Public Health Institute, Basel, Switzerland; 2 University of Basel, Basel, Switzerland; 3 Centre de Depistage et de Traitement de l'Ulcere de Buruli d'Allada, Allada, Benin; Fondation Raoul Follereau, France

## Abstract

**Background:**

While traditionally surgery has dominated the clinical management of Buruli ulcer (BU), the introduction of the combination chemotherapy with oral rifampicin and intramuscular streptomycin greatly improved treatment and reduced recurrence rates. However management of the often extensive lesions after successful specific therapy has remained a challenge, in particular in rural areas of the African countries which carry the highest burden of disease. For reasons not fully understood, wound healing is delayed in a proportion of antibiotic treated BU patients. Therefore, we have performed immunohistochemical investigations to identify markers which may be suitable to monitor wound healing progression.

**Methodology/Principal findings:**

Tissue specimens from eight BU patients with plaque lesions collected before, during and after chemotherapy were analyzed by immunohistochemistry for the presence of a set of markers associated with connective tissue neo-formation, tissue remodeling and epidermal activation. Several target proteins turned out to be suitable to monitor wound healing. While α-smooth muscle actin positive myofibroblasts were not found in untreated lesions, they emerged during the healing process. These cells produced abundant extracellular matrix proteins, such as pro-collagen 1 and tenascin and were found in fibronectin rich areas. After antibiotic treatment many cells, including myofibroblasts, revealed an activated phenotype as they showed ribosomal protein S6 phosphorylation, a marker for translation initiation. In addition, healing wounds revealed dermal tissue remodeling by apoptosis, and showed increased cytokeratin 16 expression in the epidermis.

**Conclusion/Significance:**

We have identified a set of markers that allow monitoring wound healing in antibiotic treated BU lesions by immunohistochemistry. Studies with this marker panel may help to better understand disturbances responsible for wound healing delays observed in some BU patients.

## Author Summary

Coagulative tissue necrosis and local immunosuppression caused by the *M. ulcerans* macrolide toxin mycolactone are typical features of Buruli ulcer disease (BU). In particular in BU endemic remote rural areas of West Africa, patients often report with large ulcerated lesions. Despite the availability of an effective dual antimycobacterial antibiotic therapy, some ulcerative lesions may take long time to healing and represent a major burden for the patients as well as for the health system. Proper wound healing is a well-orchestrated process involving numerous cellular and acellular components. Here we have performed immunohistochemical studies with tissue from BU lesions collected before, during and after antibiotic treatment. We identified a set of markers which are appropriate to evaluate formation of granulation tissue (alpha-smooth muscle positive fibroblasts), matrix deposition (pro-collagen 1, fibronectin and tenascin C), cell activation (phosphorylated S6), hyper proliferation of the epidermis (cytokeratin 16) and apoptosis (cleaved caspase 3) during wound healing. These markers may become suitable for assessing progression of tissue repair and for investigating the functional basis of impaired wound healing.

## Introduction

Buruli ulcer (BU) is a necrotizing skin disease caused by *M. ulcerans*. It is primarily affecting the subcutaneous tissue and can, if untreated, lead to extensive tissue destruction and ulceration. The disease has been reported from more than 30 mainly tropical countries [1] around the world with the highest incidence in West Africa. The distribution of the disease is very focal and typically associated with rural wetlands in close proximity to stagnant or slow flowing water bodies [1], [2]. The mode of transmission and the environmental reservoir of *M. ulcerans* are still not fully characterized.

The disease can affect all age groups, but the highest incidence is in children aged between 5 and 15 years and in the elderly [3], [4]. Most of the lesions occur on the limbs, but all parts of the body can be affected. The currently recommended treatment consists of daily administration of oral rifampicin (10 mg/kg) and intramuscular streptomycin (15 mg/kg) for 8 weeks under regular supervision.

BU presents with a variety of clinical forms including nodules, plaques, edema and ulcers and in more severe cases multiple lesions as well as osteomyelitis have been observed. The disease often starts as a painless swelling or an area of induration which eventually may develop the characteristic features of BU such as large ulcers with undermined edges [5]. In particular in remote areas of Africa, patients tend to report late to the treatment centers and therefore often with very extensive and severe lesions. Long recovery periods are common and in the case of large lesions skin transplantation is required and permanent morbidities including functional limitations may be observed [6]. While mycolactone causes massive local immune suppression in active BU lesions, vigorous local immune responses are observed during anti-mycobacterial chemotherapy [7]. Paradoxical reactions including the enlargement of ulcers, progression of non-ulcerated plaques and edemas to ulcerative lesions, and the emergence of new lesions are frequently observed during chemotherapy [8], [9]. However, neither ulceration of plaques [10] nor the appearance of new lesions [11], [12] is necessarily an indicator of a failure of chemotherapy. While many BU lesions tend to heal fast after completion of anti-mycobacterial treatment in some patients, the healing process is severely delayed in others. Massive infiltration of lesions and the development of atopic lymphoid tissue are usually not interfering with wound healing. Vigorous local immune responses are thus a good marker of the success of anti-mycobacterial treatment [7], but not necessarily associated with complications. In patients being treated for BU, it is therefore difficult to differentiate between deterioration resulting from a local immune reconstitution inflammatory syndrome [13] and deterioration resulting from other causes, such as disturbed orchestration of wound healing processes or secondary infections.

Wound healing is a complex process [14], consisting of a sequence of four overlapping and integrated phases: rapid homeostasis, inflammation, proliferation and tissue remodeling [15], [16] which are characterized by inter- and intra-cellular level changes. Once the vascular constriction and the fibrin clot are in place, inflammatory cells migrate into the wound bed and promote the inflammatory phase characterized by the infiltration of macrophages, lymphocytes and neutrophils which clean the wound area, release cytokines to induce inflammation and to stimulate fibroblasts, keratinocytes and other elements involved in the subsequent phase of the wound healing process [17]. The inflammatory phase is in general followed, but partly also overlapping, with the proliferative phase. The proliferative phase is characterized by epithelial proliferation and migration through a “temporal” extracellular matrix (ECM) composed of several proteins including fibronectin, tenascin and pro-collagen I. This matrix acts as support for the fibroblast migration into the wound bed. Fibroblasts and endothelial cells are abundant at this time and they support capillary growth and formation of granulation tissue. Myofibroblasts, which are specialized fibroblasts, are the main producers of collagen in healing wounds. Initially type III collagen is produced and then replaced by type I collagen [18]–[20]. Myofibroblasts can contract by using a smooth muscle type actin-myosin complex, rich in α-smooth muscle actin (αSMA) and are involved in the contraction and closure of wounds [21], [22]. αSMA is commonly used as a marker for the detection of myofibroblasts, but it is also present in pericytes located at the wall of blood vessels. After healing is complete, myofibroblasts are normally eliminated by apoptosis and in healthy tissue they are present only sub-epithelially in mucosal surfaces [15]. However, myofibroblasts seem to persist in wound granulation tissue that fails to resolve after healing, which is considered to be the cause of excessive matrix deposition in hypertrophic scars [15], [23]. The final remodeling phase of wound healing is also characterized by a reduction in the number of newly formed vessels and a slow return to conditions similar to healthy skin tissue [15]. Here we have analyzed markers of cell activation, myofibroblast formation and matrix deposition in tissue biopsies from BU lesions before, during and after treatment.

## Materials and Methods

### Ethics statement

Ethical approval (clearance N° 011, 12/10/2010) for the analysis of the clinical specimens was obtained from the provisional national ethical review board of the Ministry of Health Benin, registered under the N° IRB00006860. Tissue samples were taken for detailed immunohistological analysis, after written informed consent has been given by the patients or their guardians.

### Study participants

Eight patients from a highly endemic region of Benin (Ze commune in the Atlantique department) with laboratory confirmed BU plaque lesions which reported to the Centre de Depistage et de Traitement de l'ulcere de Buruli d'Allada, between April and August 2009 were included in the study. For all eight patients, biopsies and material obtained during wound debridement or excisions were available. Samples were taken at 3 different time points: T1 before the start of antibiotic treatment (day-2 to 0), T2 during antibiotic treatment (day 26–34) and T3 after the completion of antibiotic treatment (day 56–72). The age of patients ranged from five to 70 years and lesions were mostly (5/8) present at the lower extremities (Table 1). Clinical diagnosis of BU was reconfirmed at least with 2 of 3 laboratory tests applied (ZN staining, IS 2404 PCR and histopathology). All patients tested negative for HIV, completed the eight weeks of antibiotic treatment as recommended by the WHO and lesions were closed and healed for all patients by day 127 after completion of therapy [10].

**Table 1:** Patient cohort.

Patient Nr.	Sex	Age (years)	Site of lesion	Size of lesion	Days of R/S treatment	Sample T1 (days)	Sample T2 (days)	Sample T3 (days)
1	F	13	upper leg	9 cm×8 cm	56	−2	27	65
2	F	20	foot	5 cm×4 cm	56	−2	27	65
3	M	15	elbow	5 cm×5 cm	56	−2	26	65
4	F	70	knee	8 cm×5 cm	56	−2	27	65
5	M	32	upper leg	15 cm×4 cm	56	−2	29	68
6	M	5	lower arm	12 cm×10 cm	56	−2	26	72
7	M	12	hand	12 cm×13 cm	56	0	28	56
8	M	12	lower lag	10 cm×8 cm	56	−1	34	62

### Tissue processing and staining

Punch biopsies and tissue samples removed during surgical procedures were transferred to a 10% neutral buffered formalin solution for 24 hours. Afterwards samples were stored and transported in 70% ethanol, embedded into paraffin and cut into 5 µm sections with a microtome. Sections were recovered on glass slides and after deparaffinization stained with Haematoxylin/Eosin (HE) to obtain an overview of the tissue structure and with Ziehl-Neelsen (ZN) to detect acid-fast bacilli.

For immunohistochemical and immunofluorescence analysis tissue samples were stained with the antibodies and protocols listed in Table 2. For immunohistochemical staining the ABC and the NovaRED Kits from Vector laboratories were used and sections were counterstained with Haematoxylin. Immunofluorescence staining was performed by using secondary antibodies coupled to Alexa fluor 488 or Alexa fluor 594 and sections were counterstained with DAPI.

**Table 2:** Primary antibodies.

Name	Company	Monoclonal/Polyclonal	Host	Pre treatment	Dilution
Cytokeratin 16	Novocastra	Clone LL025	Mouse	Citrate	1∶100
Pro-Collagen I	Millipore	Clone M-58	Rat	Citrate	1∶500
αSMA	Novocastra	Clone α sm-1	Mouse	Citrate	1∶100
Tenascin	Dako	Clone TN2	Mouse	Trypsin	1∶1000
Fibronectin	Novocastra	Clone 568	Mouse	Trypsin	1∶500
Phospho-S6^235/236^	Cell Signalling	polyclonal	Rabbit	Citrate	1∶400
CC3	Cell Signalling	polyclonal	Rabbit	Citrate	1∶100

### Histopathological features of tissue biopsies

The tissue biopsies analyzed here for wound healing markers have been analyzed previously for the development of inflammatory infiltrates [10]. Shortly, before start of treatment (T1), the plaque lesions presented with an intact epidermis and dermis with relatively intact collagen and minor infiltration around glands and vessels. The subcutis appeared necrotic and edematous with fat cell ghosts. Samples taken four to five weeks after start of antibiotic treatment (T2), showed some infiltration with CD20^+^ B-cells, CD3^+^ T-cells and macrophages and early granuloma formation. Large necrotic areas were still present at this stage. After completion of antibiotic treatment (T3) large surgically excised tissue specimens comprised areas with largely healthy appearance, strongly infiltrated areas and completely necrotic areas without infiltration [10].

## Results

### Emergence of αSMA-positive, phospoS6-activated myofibroblasts in antibiotic treated BU lesions

**Figure 1:**
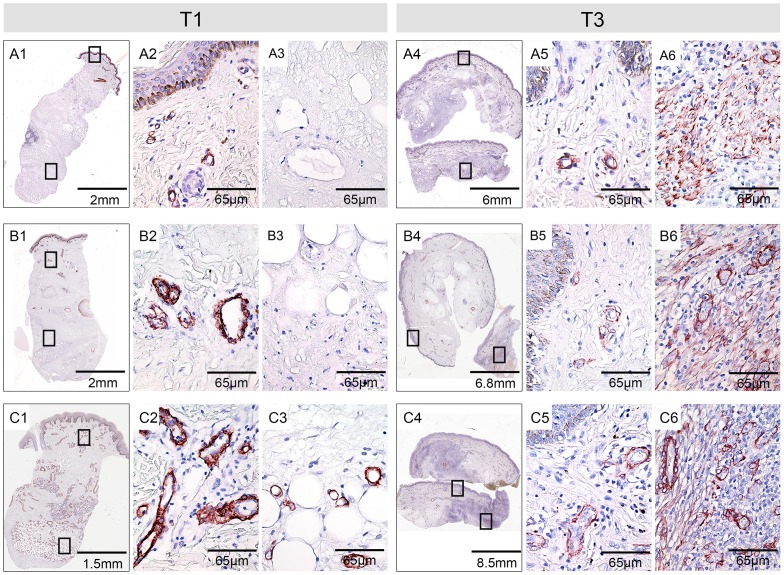
Emergence of αSMA-positive myofibroblasts in antibiotic treated BU lesions. Histological sections were stained with an anti-αSMA antibody and counterstained with Haematoxylin. Typical results with tissue specimens of three BU patients (A, B and C) are shown. Specimens were taken before or after completion of antibiotic therapy. A1, B1, C1: scans of the stained punch biopsies taken before commencement of antibiotic therapy. A2, B2, C2: higher magnification showing that αSMA staining in the dermis before treatment is restricted to blood vessels. A3, B3, C3: Higher magnification of the subcutaneous tissue showing blood vessel staining in only 1/8 patients (C3) and no specific staining in the other patients (A3, B3). A4, B4, C4: scans of the tissue excised after completion of antibiotic treatment. A5, B5, C5: αSMA-positive blood vessels were found in the dermis of all patients. A6, B6, C6: large numbers of αSMA positive myofibroblasts were found in strongly vascularized subcutaneous areas after completion of therapy.

Immunohistochemical staining for αSMA revealed staining of blood vessel walls in the dermal tissue in all eight tissue samples from BU plaque lesions collected prior to antibiotic treatment (T1) (Fig. 1 A2, B2, C2). In contrast, no staining of blood vessel walls was observed in the necrotic subcutaneous areas of 7/8 lesions (Fig. 1: A3, B3). Only 1/8 lesions (Fig. 1 C3) showed also blood vessel staining in the subcutaneous tissue before commencement of therapy. During treatment (T2) no change of the αSMA staining pattern was observed in 6/8 lesions, compared to T1. However, in 2/8 lesions, small numbers of myofibroblasts were already present at this time point in the dermis and subcutis (data not shown). After completion of antibiotic treatment (T3) an increase in αSMA staining was observed in 7/8 patients. Extensive blood vessel staining was now also found in the subcutaneous tissue, indicative for the development of new blood vessels in the previously damaged tissue areas. In addition, in 7/8 lesions αSMA-positive myofibroblasts were found in the subcutaneous tissue in association with other infiltrating cells (Fig. 1A6, B6, C6).

**Figure 2:**
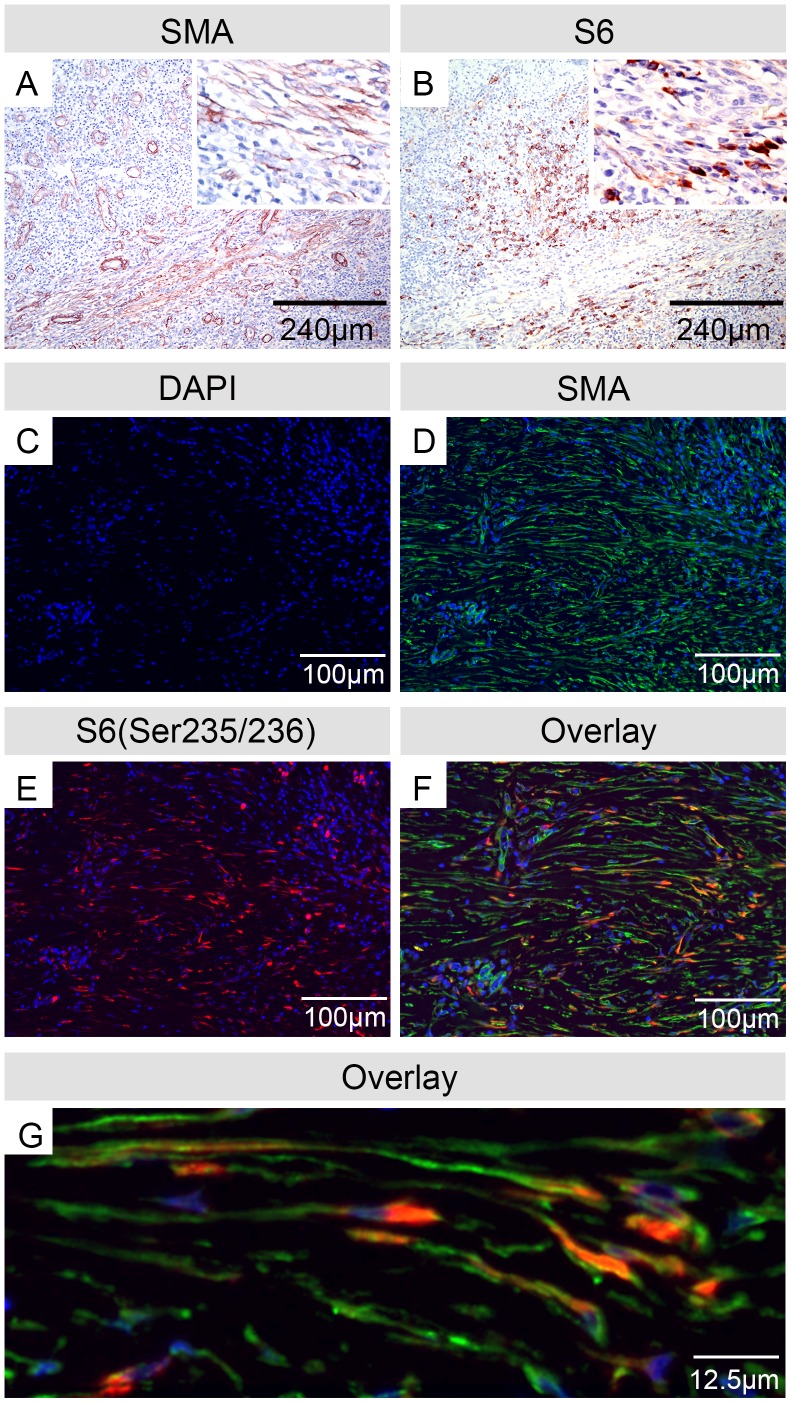
αSMA-positive myofibroblasts in antibiotic treated BU lesions show phosphorylation of the S6 ribosomal protein. Histological sections were stained either by immunohistochemistry and counterstained with Haematoxylin (A, B) or by immunofluorescence double staining (C–G) and DAPI counterstaining of nuclei (C). αSMA staining (A) and Phospho-S6^235/236^ staining (B) were found in the same tissue areas. Immunofluorescence staining for αSMA (D) and Phospho-S6^235/236^ (E) revealed co-staining of cells with fibroblast morphology (F, G), demonstrating that the mTor pathway is activated in the myofibroblasts emerging in antibiotic treated BU lesions.

Notably, myofibroblast-rich subcutaneous areas contained numerous cells, which showed phosphorylation of the S6 ribosomal protein (Fig. 2A, 2B), a well-established marker for downstream effects of mTor signaling. This prompted us to investigate whether S6 is activated in myofibroblasts of healing BU lesions. Double staining with antibodies specific for αSMA and the Serine^235/236^ phosphorylated version of the S6 protein were performed on the seven myofibroblast-containing BU tissue samples collected after completion of antibiotic treatment (T3). αSMA-positive fibroblasts revealed Phospho-S6^235/236^ staining, which indicates that the mTor pathway is activated in the myofibroblasts emerging in antibiotic treated BU lesions (Fig. 2).

### The ECM proteins tenascin, fibronectin and pro-collagen 1 are expressed in healing BU lesions

**Figure 3:**
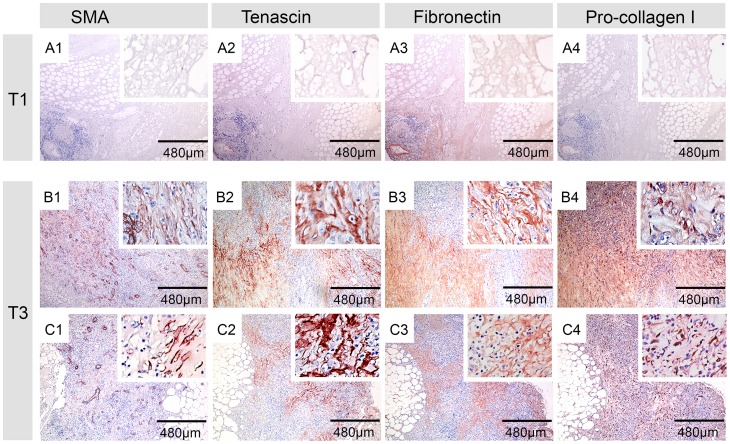
Increased expression of the ECM proteins tenascin, fibronectin and pro-collagen 1 in healing BU lesions. Serial histological sections were stained with antibodies against αSMA and the ECM proteins tenascin, fibronectin and pro-collagen 1 and counterstained with Haematoxylin. Panel A represents a typical lesion before commencement of antibiotic therapy (T1) and Panel B and C typical tissue specimens from two patients after completion of therapy (T3). Whereas no or only weak staining for αSMA, tenascin, fibronectin and pro-collagen 1 was observed before therapy (A1–A4), tissues turned strongly positive for all four markers after completion of treatment (B1–B4 and C1–C4). Staining of ECM proteins was most prominent in areas containing many αSMA positive myofibroblasts.

Besides αSMA also the expression and distribution of the ECM proteins tenascin, fibronectin and pro-collagen 1 turned out to reflect progression to wound healing (Fig. 3). In tissue samples from untreated BU patients (T1), only weak cellular and/or subcellular staining of fibronectin and tenascin was observed primarily in the dermal region (Fig. 3 A2, A3). The amount of these proteins increased after completion of therapy (T3) in all eight patients, as shown in Fig. 3 for two typical patient samples (B and C). The increase in these two proteins was most evident in subcutaneous areas, where also the αSMA-positive myofibroblasts emerged.

Before treatment (T1) only few cells, primarily located in the dermal tissue layer, expressed pro-collagen 1 (Fig. 3 A4). After treatment (T3) substantial numbers of pro-collagen 1-positive cells were detected all over the tissue specimen (Fig. 3 B4, C4). These were particularly abundant in areas where αSMA-positive myofibroblasts were present (Fig. 3 B1, B2), suggesting that myofibroblasts are a major source of newly synthesized pro-collagen 1 in healing BU lesions.

### Enhancement of cytokeratin 16 expression in keratinocytes early after commencement of BU treatment

**Figure 4:**
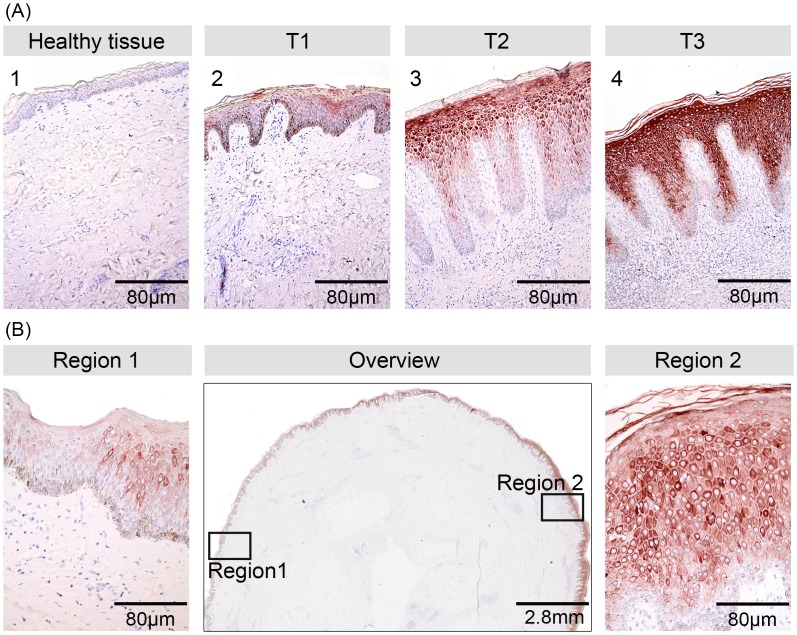
Increase of Cytokeratin 16 expression by keratinocytes during antibiotic therapy. Histological sections were stained by immunohistochemistry with an anti-Cytokeratin 16 antibodies and were counterstained with Haematoxylin. While healthy skin was completely devoid of Cytokeratin 16 staining (A1), some staining was observed (A2) in the epidermal layer of untreated BU lesions (T1). Staining intensity and epidermal thickness increased in samples collected during (T2) and after completion (T3) of antibiotic therapy (A3, A4). After completion of therapy (T3) heterogeneous staining (B, Overview), with some areas of the epidermal layer showing much weaker Cytokeratin 16 staining (Region 1) than others (Region 2) was observed.

Cytokeratin (CK16) expression in the epidermis is a marker for keratinocyte hyper-proliferation [24], as found associated with wound healing. It is not present in healthy epidermal skin (Fig. 4 A1). Epidermal hyper proliferation is a characteristic feature in BU lesions, and in 4/8 lesions collected prior to treatment some CK16 staining of keratinocytes was observed (Fig. 4 A2). CK16 staining increased in intensity and extension during (Fig. 4 A3) and in particular after completion of antibiotic treatment (Fig. 4 A4). Along with this, epidermal hyperplasia was also more pronounced after therapy in 7/8 patients. Another characteristic observed for CK16 was the heterogeneity of the intensity of staining within individual specimen (Fig. 4B, Region1, Region 2), which may reflect diversity in keratinocyte activation in different areas of BU lesions.

### Emergence of apoptotic fat cells after therapy may reflect tissue remodeling

**Figure 5:**
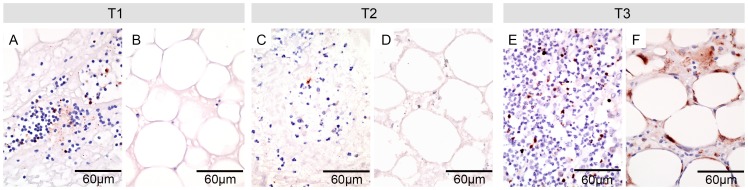
Emergence of apoptotic fat cells after completion of antibiotic therapy. Histological sections were stained by immunohistochemistry with anti-CC3 antibodies and were counterstained with Haematoxylin. Infiltrated necrotic areas (A, C, E) and fat cell layers (B, D, F) of the subcutaneous tissues are displayed. Before treatment some of the infiltrating cells showed CC3 staining (A). No staining was observed in the subcutaneous layer (B). During treatment (C, D) only very few cells showed CC3 staining. After treatment substantial numbers of infiltrating cells were CC3-positive (E) and in addition larger numbers of CC3-positive fat cells were found (F).

Caspase 3 is a main effector caspase of the apoptotic cascade and antibodies specific for neoantigens of cleaved caspase 3 (CC3) are a useful tool to identify apoptotic cells in paraffin embedded tissue. For all BU lesions we observed a decrease of CC3-positive cells during treatment (T2) and an increase after therapy (T3) (Fig. 5). Before commencement of antibiotic therapy CC3-positive cells were observed in very small numbers in infiltrated areas of intact dermal tissue and around remaining blood vessels (Fig. 5A). The necrotic areas were devoid of CC3-positive cells, but contained abundant numbers of fat cell ghosts, which may have already gone through apoptosis (Fig. 5B). During treatment CC3-positive cells were only very rarely observed in the dermal and subcutaneous layer (Fig. 5C, D). After completion of treatment some of the infiltrating immune cells were CC3-positive (Fig. 5E), which may reflect physiological elimination of inflammatory cells. Notably, 6/8 patients revealed strong CC3 staining of fat cells far away from the necrotic core (Fig. 5F), which suggests that apoptosis of fat cells may be an element of tissue remodeling in healing BU lesions.

## Discussion

Specific treatment of BU is highly effective since the introduction of the R/S antibiotic combination therapy in 2004 and recurrence rates could be reduced [8]. Beside this promising development wound healing and wound management is still a major problem in the remote rural BU endemic areas of Africa where patients tend to present late to hospitals [25]. While the mycobacteria may be efficiently eliminated by the specific treatment, large open wounds may persist and often prolonged wound care and skin grafting are necessary [26], [27]. Why some BU lesions heal very fast while others require a long time till complete healing, is unclear and not related to the size or lesion type. Paradoxical reactions may be caused by secondary bacterial infections [28], immune reconstitution inflammatory syndrome like mechanisms [11], [13], [29], [30] or inappropriate cell activation and disturbed transition from the inflammatory to the healing phase. For future characterization of mechanisms causing wound healing delays, we have studied here the emergence and spatial distribution of wound healing markers in healing BU lesions.

Important key players during wound healing are αSMA-positive myofibroblasts. While in healthy skin αSMA is typically present only in cells located at the walls of blood vessels and in skin adnexa, it is also produced in healing wounds by myofibroblasts, a highly specialized cell type involved in granulation tissue formation, production of ECM proteins, and wound contraction [23]. After completion of antibiotic treatment we observed these specialized fibroblasts in substantial numbers in regenerating BU lesions. Minimal presence of myofibroblasts during the treatment phase (T2) is probably related to the massive mycolactone mediated tissue necrosis which delays granulation tissue formation.

Further analysis revealed that these myofibroblasts were activated via the intracellular regulatory mTor pathway, which mediates cellular events critical in cell proliferation, movement and metabolism [31], [32]. In mice PI3K-Akt activation promotes cutaneous wound repair and an elevated mTor activity strongly accelerates wound healing [33].

Therefore it is speculated that activation of this pathway in humans might help to treat large, chronic and life threatening wounds and accelerate wound healing [34]. Antibodies binding to the phosphorylated S6 protein can be used to determine whether cells show an activation of the mTor pathway [32], [35]. Here we observed that, after treatment of BU lesions, many cells, most notably αSMA-positive myofibroblasts, exhibited increased S6 phosphorylation, indicating enhanced protein synthesis and/or proliferation. In contrast, phosphorylated S6 protein positive cells are barely detectable in healthy skin [36].

Malnutrition or starvation is known to repress mTor activation (most likely via Insulin/IGF deficiency) [35] and may contribute to impaired wound healing in some BU patients. Based on these results, we will investigate in a next step whether mTor pathway activation can help to discriminate between healing and non-healing wounds in BU.

Activated myofibroblasts are not only involved in wound contraction but they also produce the ECM proteins fibronectin, tenascin and different pro-collagens. Together with newly formed blood vessels and inflammatory cells they built the granulation tissue, which is a prerequisite for successful healing of dermal injuries [15]. In healthy skin, fibronectin is found in blood vessels, in dermal/epidermal junctions and in hair follicles [17], [20]. In contrast tenascin-C and pro-collagen 1, which is the zymogen of collagen 1, are almost absent in the healthy skin but are abundant in healing skin lesions [37]–[39]. The presence of activated myofibroblasts and deposition of ECM proteins in response to BU specific therapy is a strong indication for a successful ongoing wound healing process. We observed the formation of granulation tissue in all 8 patients after completion of therapy and in 2/8 patients already at time point T2 during chemotherapy. Indeed all BU lesions analyzed in this study, showed a good clinical outcome, no recurrences were observed and patients could be discharged from the hospital 42 to 127 days after completion of therapy [10].

Although, the epidermis and dermis of plaque lesions may stay closed and intact for a long time [10], the expression of CK16 is a clear indication that also the epidermis is affected in BU lesion. CK16 is a marker of epidermal hyper-proliferation and is expressed by activated keratinocytes in wounded tissue [24]. In antibiotic treated BU lesions we observed an increase of CK16 expression over time. Absence or only faint CK16 staining in tissue taken before therapy, support the idea that the cytotoxic and immunosuppressive effects of mycolactone, which arrests the lesions in a chronic wound healing state and also suppresses keratinocyte activation. Heterogeneous staining of the epidermal layer in larger surgical excisions excised at time point T3 may reflect disease activity in the underlying tissue. Augmented epidermal CK16 expression is also characteristic for inflammatory skin diseases with a hyper-proliferative epidermis, such as psoriasis, and CK16 is used as marker to evaluate the efficacy of anti-psoriatic treatments [40]. In addition, intra-dermal injection of the pro-inflammatory cytokine interferon-gamma has been shown to increase epidermal CK16 expression [41]. Therefore, enhanced CK16 expression in BU lesions after treatment is likely the consequence of increased dermal inflammation in response to successful antibiotic treatment and mycolactone clearing.

Each phase of the complex wound healing process is characterized by the presence of a specific population of cells producing specific proteins and fulfilling specific tasks. Tissue remodeling by controlled cell death (apoptosis) is as important as proliferation. If cell death is wrongly regulated and certain cells persist after their task is accomplished this may lead to wound healing complications, like the development of hypertrophic scars or keloids [23], [42]. Additionally, infiltrating inflammatory cells need to be removed after the wounded area has been cleaned.

Here we used CC3 as an established marker which is detectable in a small time window in end-stage apoptotic cells. Its short duration of expression explains the small number of positive cells in our BU lesions. It is known that mycolactone induces apoptosis in vitro and in vivo leaving behind only necrotic tissue devoid of any surviving cells [43], [44]. Accordingly, at time point T1, subcutaneous tissue presented nearly devoid of any intact cell, except for some remaining cells around blood vessels and CC3 positive cells were very rare. While a further decrease of CC3 staining was observed during antibiotic treatment (T2), after treatment (T3) CC3 staining became prominent in the granulation tissue and was even more pronounced in nearby fat tissue. At this stage the wound healing process is in a state between the inflammatory and the remodeling phase. The inflammatory phase sets in shortly after the start of treatment and is characterized by a strong mixed infiltration, presence of granulomas and giant cells as well as B-cell cluster [30]. Apoptosis of inflammatory cells observed in the temporary granulation tissue at time point T3 is a necessary step in order to form healthy new tissue [15]. No apoptotic myofibroblasts were detected, since they are still needed at this stage of the wound healing process. The observed emergence of apoptotic fat cells may also be a result of the ongoing tissue remodeling process and reflect the removal of superfluous or impaired fat tissue. In conclusion this study shows that markers like αSMA, fibronectin, pro-collagen 1, tenascin-C and CK16, are suitable to monitor healing of BU lesions. The present study also suggests that the mTor pathway might play an important role during wound healing in BU. Further investigations using the presented marker set to compare healing and non-healing BU lesions may help to clarify steps and aspects of this complex process. Whether wound healing deficiencies are associated with insufficient activation of the mTor pathway needs to be examined in a larger cohort.
